# Factors Associated With Depressive Symptoms in Female Victims of Intimate Partner Violence in Southern Taiwan

**DOI:** 10.1097/jnr.0000000000000303

**Published:** 2019-07-16

**Authors:** Hsiu-Fen Hsieh, Bih-Ching Shu

**Affiliations:** 1 PhD, RN, Assistant Professor, College of Nursing, Kaohsiung Medical University, and Adjunct Research Fellow, Department of Medical Research, Kaohsiung Medical University Hospital, Kaohsiung Medical University; 2 PhD, RN, Distinguished Professor, Institute of Allied Health Sciences and Department of Nursing, College of Medicine, National Cheng Kung University.

**Keywords:** intimate partner violence, physically abused women, personality, depressive symptoms

## Abstract

**Background:**

Intimate partner violence (IPV) is known to cause physical suffering and psychological problems, which burden society. In addition, IPV-related psychological problems such as depressive symptoms may lead to disabilities, chronic mental illness, and an increased risk of suicide.

**Purpose:**

This study was designed to explore the factors associated with depressive symptoms in women who were physically abused by intimate partners.

**Methods:**

This cross-sectional study recruited 72 physically abused women from two domestic violence prevention centers in southern Taiwan. The questionnaires that were used to collect data included the Eysenck Personality Questionnaire, the Conflict Tactic Scale, and the Center for Epidemiologic Studies Depression Scale.

**Results:**

Sixty-six of the participants (91.67%) met the inclusion criteria and completed all questionnaires. Fifty-five (83.3%) of the participants were found to have depressive symptoms. Depressive symptoms were positively associated in this sample with younger age, a lower level of extraversion, and a higher level of neuroticism. These three factors explained 59.5% of the total variance in depressive symptoms.

**Conclusions/Implications for Practice:**

Youth and neuroticism were found to be significant risk factors for developing depressive symptoms in female victims of IPV, whereas extraversion was found to be a related protective factor. The results of this study indicate that clinical workers should provide female victims of IPV, especially relatively young victims, with services that help ameliorate neuroticism to reduce the risk of depressive symptoms.

## Introduction

Intimate partner violence (IPV) against women is a widespread social problem and a serious challenge to human rights that severely affects public health. The prevalence of physical IPV worldwide reportedly ranges from 13% to 61% (World Health Organization [WHO], 2012). IPV is perpetrated by a current or ex-spouse, cohabitant, or regular partner (United Nations, 2010). The potential causes of IPV are myriad, and IPV incidents may be explained by individual, relationship, community, and societal factors. Specifically, low level of education, personality disorders, economic stresses, relationship conflicts, and alcohol consumption have been identified as factors that increase the risk of IPV (Jewkes, 2002; WHO, 2012).

The number of victims of IPV has increased in recent years in many countries (WHO, 2012), including Taiwan. According to official statistics from the Taiwan Ministry of Health and Welfare, the number of victims of IPV has nearly doubled, from 79,874 cases in 2008 to 117,550 cases in 2016; 80% of victims are female (Ministry of Health and Welfare, Taiwan, ROC, 2017). IPV is often undetected or underreported because of cultural or economic factors. Some victims are influenced by the value of “don't wash your dirty linen in public” (Yang, 1992), whereas others are reticent to seek help because of economic dependence on their abusers. A survey of IPV victims found that nearly half chose to stay in the relationship and to tolerate their situation to protect their children (Pan & Yu, 2012).

The health consequences of IPV have been well documented. A systematic review of 13 studies showed a strong association between IPV and factors such as depression and posttraumatic stress disorder and found physical violence to be the strongest predictor of poor mental health (Chmielowska & Fuhr, 2017). Mental health problems are a risk factor for disability, chronic mental illness, and suicide (Walton-Moss, Manganello, Frye, & Campbell, 2005; WHO, 2005).

Furthermore, the association between IPV and depressive symptoms has been well documented in numerous other studies (Gong et al., 2014; Karakuła Juchnowicz, Łukasik, Morylowska-Topolska, & Krukow, 2017; Meekers, Pallin, & Hutchinson, 2013). Help-seeking behavior has been associated with the severity and frequency of violence (Ergöçmen, Yüksel-Kaptanoğlu, & Jansen, 2013), and severity of violence has been positively associated with the severity of depressive symptoms (Ansara & Hindin, 2011; Avdibegović & Sinanović, 2006).

Personality traits have been identified as possible key factors that influence psychological health (Thomas, Raynor, & Ribott, 2015). Moreover, the personality trait of neuroticism has been identified in multiple studies as the strongest factor associated with depressive symptoms (Ross, 2011; Ulloa, Hammett, O'Neal, Lydston, & Leon Aramburo, 2016). Previous studies have positively associated degree of neuroticism with severity of depressive symptoms and found that neuroticism has a strong impact on quality of life (Ross, 2011; Ulloa et al., 2016). In contrast, degree of extraversion has been positively associated with the ability to deal with negative emotions and to seek out social resources (Campbell-Sills, Cohan, & Stein, 2006). Furthermore, a review article covering 37 relevant studies concluded that a positive association exists between IPV and depression (Beydoun, Beydoun, Kaufman, Lo, & Zonderman, 2012).

Social and cultural differences exist between Taiwan and other countries (Chen & Yo, 2001), and more attention should be paid to the mental health of women. For these reasons, this study attempts to identify the factors associated with depressive symptoms in female victims of IPV to provide a reference for developing new and improving existing interventions to protect the mental health of this vulnerable population.

Therefore, this study aimed to (a) explore the associations among severity of physical violence, personality factors, and depressive symptoms in a sample of female IPV victims in Taiwan and (b) identify factors associated with the development of depressive symptoms.

## Methods

### Design and Sample

A cross-sectional study was conducted using questionnaires. A convenience sampling technique was used to recruit female victims of IPV who voluntarily sought legal advice from the Domestic Violence and Sexual Assault Prevention Center (DVSAPC) of a city in southern Taiwan. DVSAPC social workers met with the individuals, provided information about this study, and then introduced the first author, who explained the study purpose. Face-to-face interviews were conducted by the first author to establish rapport with participants before data collection.

The minimum sample size for this study, calculated using G*Power 3.1.1 version (Heinrich Heine Universitat, Dusseldorf, Germany) with an effect size of .35, a desired significance level of .05, and a power of .80 (Kling, Hyde, Showers, & Buswell, 1999), was estimated to be 67. We recruited potential participants from the DVSAPC who met the following inclusion criteria: (a) experienced physical violence inflicted by their intimate partners, (b) at least 18 years old, and (c) able to speak Mandarin or Taiwanese. Otherwise qualified individuals were excluded if they were unable to provide informed consent or had a diagnosis of mental disorder before or after experiencing physical violence.

### Ethical Considerations

After obtaining approval from the institutional review board (ER-9605), the principal investigator informed all participants that they could withdraw from the study at any time without giving any reason. To ensure that the participants felt safe and comfortable, each was interviewed individually in a private, secure room. All identifying information was anonymized, and all information obtained in connection with this study was held in full confidentiality.

### Procedures

Data were collected using individual, structured interviews. The principal researcher distributed, illustrated, and explained the consent forms and questionnaires to every participant, who completed the forms independently. Participation in the study was wholly voluntary. Data collected included demographic characteristics (age, level of education, marital status, and religious affiliation), the Eysenck Personality Questionnaire (EPQ), the Conflict Tactics Scale, and the Center for Epidemiologic Studies Depression Scale (CES-D).

### Personality

The EPQ Chinese Version (Lu, 1994) contains 25 items measuring neuroticism, with a total possible score ranging from 0 to 14, and extraversion, with a total possible score from 0 to 11. Higher scores in the two dimensions represent higher degrees of neuroticism or extraversion, respectively. The Cronbach's alpha scores were .90 overall, .80 for the neuroticism dimension, and .83 for the extraversion dimension (Lu, 1994). Furthermore, the Cronbach's alphas for the overall EPQ, neuroticism, and extraversion subscales were .70, .82, and .81, respectively.

### Severity of Physical Violence

The Conflict Tactics Scale assesses severity of physical violence (Lin & Shen, 2003), using 10 items in two components, frequency and pattern of violence. A 7-point Likert scale was used to rate the self-reported frequency of being physically abused, with 0 = *never in the past year*, 1 = *one time in the past year*, 2 = *two times in the past year*, 3 = *three to five times in the past year*, 4 = *six to ten times in the past year*, 5 = *eleven to twenty times in the past year*, and 6 = *more than twenty times in the past year*.

The pattern of violence score is calculated as follows: throwing things (1); pushing, grabbing, or jostling (2); slapping face (3); kicking, biting, or burning with cigarette (4); hitting you with something or tying hands or feet (5); punching or kicking (6); and threatening with a weapon (7). After adding up the whole weighted scores, scores between 0 and 28 were designated as “mild conflict,” scores between 29 and 64 were designated as “moderate conflict,” and scores over 64 were designated as “severe conflict” (Lin & Shen, 2003). The Cronbach's alpha was previously determined as .90 and, in this study, as .89.

### Depressive Symptoms

The Chinese version of the CES-D evaluates the severity of depressive symptoms (Chien & Cheng, 1985). The total score of the CES-D ranges from 0 to 60, with higher scores indicating greater severity of depressive symptoms. Furthermore, scores over 15 indicate significant depression, with a Cronbach's alpha of .86. The Cronbach's alpha of the CES-D in this study was .91.

### Data Analysis

Collected data were analyzed using IBM SPSS Statistics Version 19.0 (IBM, Armonk, NY, USA). Descriptive statistics were used for the demographic characteristics and personality types of participants, duration of time spent living with an abusive partner, frequency of being abused, severity of physical violence, and depressive symptoms. Pearson's correlation coefficient was used to analyze the relationship among depressive symptoms, personality type, duration of time spent living with the abuser, frequency of being abused, and severity of physical violence. Bivariate analyses were used to identify factors that were significantly associated with depressive symptoms (*p* < .05), and these factors were entered simultaneously into the multivariate linear regression model. Because the distribution of the depressive symptom scores was skewed, the scores were log-transformed to fit the multiple linear regression model for analysis with normal distribution and the log-transformed betas were used to report associations.

## Results

Seventy-two women were invited to participate, and 66 provided complete and valid data. Five refused to participate, and one dropped out. The mean age of the participants was 32.2 (*SD* = 9.9) years. Most had been educated to the senior high school level or above, nearly two thirds were single or divorced, and roughly half were employed. More than three quarters of the participants held religious beliefs. The participants had lived with an abusive partner for a mean of 14.4 (*SD* = 9.8) years and reported durations of being battered between 0.8 and 37 years. Fifty-five (83.3%) participants self-reported experiencing depressive symptoms. The severity of physical violence was assessed using the Chinese version of the brief Conflict Tactics Scale, and the mean severity of physical violence of the participants was 71.24 (*SD* = 66.7; Table 1).

**TABLE 1.**
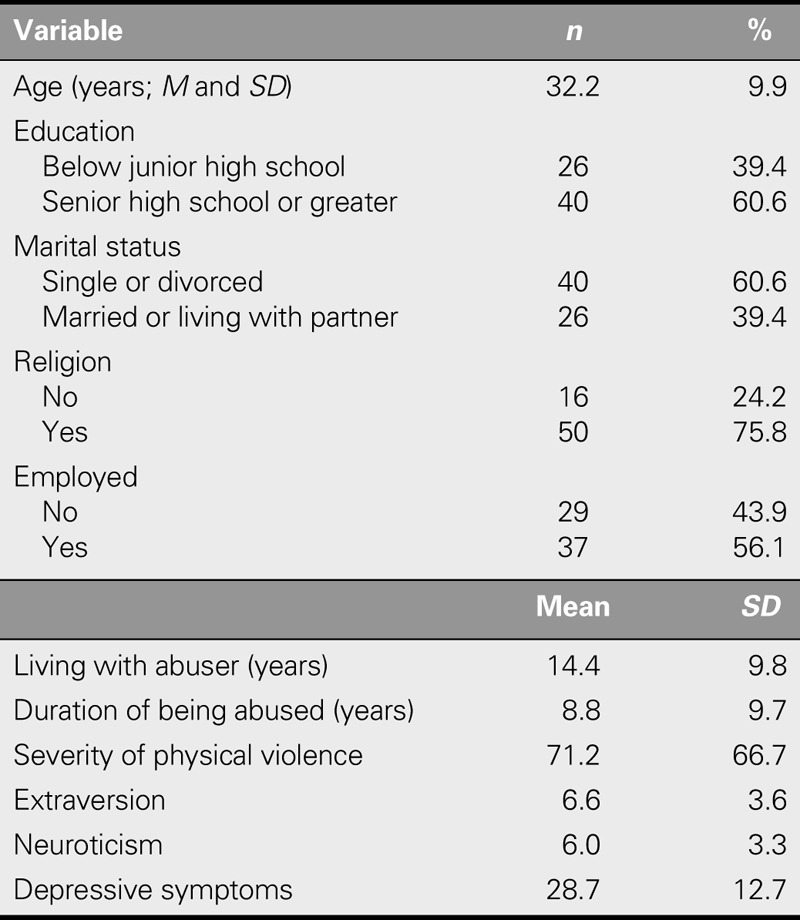
Demographic Characteristics and Measured Variables of Participants (*N* = 66)

Pearson correlation analysis was used to examine the correlations among depressive symptoms, duration of time spent living with an abusive partner, frequency of being abused, extroversion, neuroticism, and severity of physical violence. Extraversion was found to negatively correlate with depressive symptoms (*r* = −.417, *p* < .01), and neuroticism was found to positively correlate with depressive symptoms (*r* = .671, *p* < .01; Table 2).

**TABLE 2.**
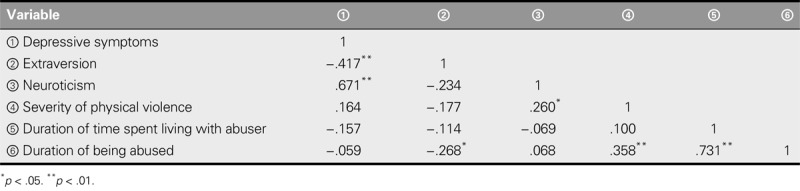
Correlations Among Depressive Symptoms, Duration of Time Spent Living With Abuser, Frequency of Being Abused, Extroversion, Neuroticism, and Severity of Physical Violence (*N* = 66)

Multiple linear regression examined the factors associated with depressive symptoms. Demographic characteristics and personality traits were entered as independent variables, and 59.5% of the variance in depressive symptoms was explained by age, extraversion, and neuroticism (Table 3). Higher degrees of neuroticism, lower degrees of extraversion, and younger age were identified as predictors of more severe depressive symptoms.

**TABLE 3.**
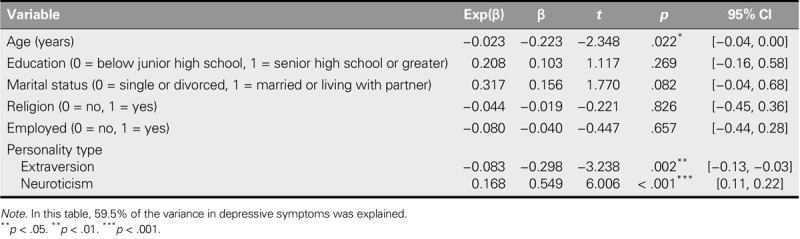
Multiple Linear Regression Analyses for Predictive Factors of Depressive Symptoms (*N* = 66)

## Discussion

Among the 66 physically abused female participants in this study, 55 (83.3%) self-reported as exhibiting depressive symptoms. This rate is higher than those found in studies from other countries, which found that approximately 74% of female victims of IPV experience depressive symptoms (Meekers et al., 2013). Using in-depth interviews, this study found that most of these participants proactively sought help from the DVSAPC because of their intractable psychological pain with depressive symptoms. This may be a major reason the participants in this study had a higher rate of depressive symptoms than those in other studies (Meekers et al., 2013; Peltzer & Pengpid, 2017). IPV has been regarded as a social norm and a “household duty” within the context of traditional Chinese culture for centuries (Chiou & Yeh, 2014). For this reason, women are greatly affected by social attitudes and are asked to be tolerant after being abused by their partners (Chiou & Yeh, 2014). Long-term, violent circumstances may be another underlying cause of depression in female IPV victims.

Another possible reason that the victims of domestic abuse develop depressive symptoms is sustained verbal threats and/or aggressive behaviors. Among the participants, 40 (60.6%) stated that they had left their violent intimate partners but still worried about further intimidating or injurious behaviors from them. In Taiwan, a “protection order” merely requests that the IPV offender keeps a “safe” distance from his or her victim, with relevant penalties for infractions of the order, and cannot absolutely prevent violence or threats (Kamimura, Ganta, Myers, & Thomas, 2014).

The finding that younger victims were more likely to have depressive symptoms than older victims echoes the results of previous studies (Jamali & Javadpour, 2016; Van Ouytsel, Ponnet, & Walrave, 2017). Owing to traditional cultural influences, older women tended to adopt “fatalism” and accept violent situations with fewer depressive symptoms (Sun, 2011). In addition, somatic complaints have been the major presentations of depressive symptoms reported by older women (Gottfries, 1998; Lapid & Rummans, 2003). The relationship between severity or frequency of physical violence and depressive symptoms was not significant in this study, which differs from prior reports (Meekers et al., 2013). On the basis of the face-to-face interview, the participants voluntarily came to the center for counseling on how to apply for protection orders, initiate divorce proceedings, and obtain custody of their children. All of these issues were more important to them than being a victim of violence. The participants did not focus on the severity of the violence. Second, most of the participants had already left their relationships and thus faced many related stressors such as loss of economic support and need to raise their children. These stressors had deeper and longer effects on the participants than the severity or frequency of violence that they had experienced. They paid significantly more attention to how they would live in the future than on the violent events that they had experienced in the past.

In addition, this study found personality to be a strong factor driving the development of depressive symptoms. Participants with higher levels of neuroticism or lower levels of extraversion faced higher risks of experiencing depressive symptoms. This result is similar to previous studies (Kendler & Myers, 2010; Knyazev, Bocharov, Savostyanov, & Slobodskoy-Plusnin, 2015). Having a relatively high level of extraversion imparts higher resilience, which helps individuals seek and acquire resources more successfully. On the contrary, a relatively high level of neuroticism increases susceptibility to negative emotions, which decreases resilience, increases neuroticism, and increases the risk of experiencing depressive symptoms, especially in the face of negative life events. IPV is a significant and negative life event for women, which may explain why personality was a factor strongly associated with the development of depressive symptoms among study participants.

### Limitations

This study is affected by several limitations. First, the cross-sectional, self-report design introduced the potential for recall bias. Second, the sample size was relatively small, and the nonresponse rate was not insignificant. Third, the number of domestic abuse victims who did not seek help from the DVSAPC is unknown. Finally, although social support and social resources have been identified in the literature as preventing the development of depressive symptoms, these variables were not measured in this study. Social support and social resources should be evaluated in related studies in the future. Moreover, further studies are needed to address the limitations previously discussed and to gain a deeper understanding of this important issue.

### Conclusions/Implications for Practice

The results of this study indicate that higher levels of neuroticism and lower levels of extraversion, respectively, predict more severe depressive symptoms in women victims of IPV. We suggest that government and healthcare providers offer assistance to combat neuroticism, such as assertiveness training, which was found to be effective. Additional factors that protect against neuroticism should be identified and incorporated into effective interventions for domestic abuse victims, especially those in younger age groups, to reduce their depressive symptoms.
